# Increase in membrane surface expression and phosphorylation of TRPC3 related to olfactory dysfunction in α‐synuclein transgenic mice

**DOI:** 10.1111/jcmm.17524

**Published:** 2022-08-27

**Authors:** Min Chen, Jia Liu, Hanjiang Luo, Chunli Duan, Ge Gao, Hui Yang

**Affiliations:** ^1^ Department of Neurobiology School of Basic Medical Sciences, Key Laboratory of Neural Regeneration and Repair, Center for Parkinson's Disease, Key Laboratory for Neurodegenerative Diseases of the Ministry of Education Beijing Institute for Brain Disorders, Capital Medical University Beijing China; ^2^ Guangxi Neurological Disease Clinical Research Center, Laboratory of Neuroscience Affiliated Hospital of Guilin Medical University Guilin China

**Keywords:** dysosmia, olfactory neurons, Parkinson's disease, TRPC3, α‐Synuclein

## Abstract

Olfactory impairment is an initial non‐motor symptom of Parkinson's disease that causes the deposition of aggregated α‐synuclein (α‐syn) in olfactory neurons. Transient receptor potential canonical (TRPC) channels are a diverse group of non‐selective Ca^2+^ entry channels involved in the progression or pathogenesis of PD via Ca^2+^ homeostatic regulation. However, the relationship between TRPC and α‐syn pathology in an olfactory system remains unclear. To address this issue, we assessed the olfactory function in α‐syn transgenic mice. In contrast with control mice, the transgenic mice exhibited impaired olfaction, TRPC3 activation and apoptotic neuronal cell death in the olfactory system. Similar results were observed in primary cultures of olfactory neurons, that is TRPC3 activation, increasing intracellular Ca^2+^ concentration and apoptotic cell death in the α‐syn‐overexpressed neurons. These changes were significantly attenuated by TRPC3 knockdown. Therefore, our findings suggest that TRPC3 activation and calcium dyshomeostasis play a key role in α‐syn‐induced olfactory dysfunction in mice.

## 1 INTRODUCTION

Parkinson's disease (PD) is characterized by motor symptoms accompanied by non‐motor symptoms, including olfactory dysfunction and disordered sleep.^1^ Among these, olfactory dysfunction is an early manifestation of motor symptoms in approximately 96% of patients 10 to 20 years before they develop motor symptoms.^2^ Post‐mortem examination of PD patient brains indicates that the olfactory bulb (OB) is one of the first regions affected by Lewy bodies (LBs), pathological hallmarks of PD. This confirms a relationship between Lewy pathology and olfactory impairment.^3^ Fibrotic α‐synuclein (α‐syn) is a significant component of LBs. α‐Syn is normally a soluble synaptic protein found in normal neurons but toxic in aggregate form.^4^ α‐Syn aggregates have been detected in the OB^5^ and olfactory epithelium (OE)^6^ of PD patients. Additionally, olfactory dysfunction has been detected in *α‐Syn* transgenic (Tg) mice, implying an association between abnormal α‐syn accumulation and olfactory impairment.^7^ Nonetheless, this relationship remains unclear.

Increased intracellular Ca^2+^ concentration is a prevalent manifestation of olfactory abnormalities^8^, ^9^ and neuronal injury.^10^, ^11^, ^12^ Of note, transient receptor potential canonical (TRPC) channels are a diverse group of non‐selective Ca^2+^ entry channels involved in sensory transduction.^13^, ^14^ TRPC family members have been implicated in PD progression via Ca^2+^ homeostatic regulation.^15^ TRPC3 is highly expressed in the nervous system^16^, ^17^ and is thought to impair dopaminergic neurons by increasing intracellular Ca^2+^ concentration.^18^ Previous reports indicate that Src tyrosine kinase, an upstream modulator of TRPC3,^19^ is activated by α‐syn overexpression.^20^ Also, we found that TRPC3 is involved in aged‐dependent α‐syn accumulation in the brains of mice and monkeys.^21^ We hypothesized that abnormal TRPC3 activity is involved in the α‐syn‐induced pathogenesis of PD. Considering that the olfactory system is a brain region affected by Lewy pathology at the early stages of PD and expresses high α‐syn levels, investigating the relationship between TRPC3 and α‐syn explains the molecular basis for olfactory dysfunction in PD.

In the present study, we discovered TRPC3 activation in OB and OE of *α‐syn* Tg mice expressing human WT *α‐syn* under the murine Thy1 (*mThy1*) promoter, accompanied by olfactory dysfunction. Similarly, in primary olfactory neurons, α‐syn overexpression induced TRPC3 activation, further increasing intracellular Ca^2+^ concentration and apoptosis. Furthermore, *TRPC3* knockdown significantly rescues the above injuries. Therefore, our findings imply that TRPC3 activation is a crucial step in the olfactory dysfunction caused by α‐syn overexpression in mice.

## 2 MATERIALS AND METHODS

### 2.1 Animals

Male Tg mice expressing human WT *α‐syn* under control of the *mThy1* promoter (line 61; Jackson Laboratories) were kept on a C57BL/6‐DBA/2 background.^22^ Animals were housed at room temperature (22–25°C) under a 12:12‐h light/dark cycle. Animal husbandry and experimental protocols adhered to the National Institutes of Health guidelines for animal care and use (NIH Publications No. 85‐23, revised 1996).

### 2.2 Lentivirus (LV)

Lentivirus gene transfer vectors encoding short hairpin (sh) RNAs (LV‐sh‐*TRPC3*, 5′‐CCA CCA AAG CGC AGC AGT A‐3′) targeting specific regions of mouse *TRPC3* mRNA and scrambled negative control (LV‐sh‐scramble) were synthesized by Genechem (Shanghai, China).

### 2.3 Reagents

Human α‐syn (hα‐syn) was detected with a mouse monoclonal antibody (3D5; a gift from Prof. Shun Yu at Xuanwu Hospital of Capital Medical University, Beijing, China).^23^ Antibodies used in this study included TRPC3 (1:1000) and TRPC6 (1:500) (Alomone Lab, Jerusalem, Israel); tyrosine hydroxylase (TH) (1:2000), β‐actin (1:2000) and glyceraldehyde 3‐phosphate dehydrogenase (GAPDH; 1:2000) (Sigma); mouse α‐syn (1:1000), caspase‐3 (1:1000) and calnexin (1:1000) (Cell Signalling Technology, Danvers, MA, USA); olfactory marker protein (Omp) (1:10,000; Wako Pure Chemical Industries, Osaka, Japan); and phosphorylated (p‐)Tyr (1:200, Santa Cruz Biotechnology). Regents used for primary neuronal culture included poly‐l‐lysine (Sigma), Neurobasal‐A medium, 50 × B27 supplement, 0.5 mM l‐glutamine (Gibco) and 100‐units/ml penicillin/streptomycin (Gibco).

### 2.4 Rotarod test

Balance and motor impairment were assessed via the rotarod test.^24^ Mice were placed on a flat, rotating rod accelerating from 5 to 35 rpm for 3 min. The time it took for each mouse to fall off was recorded as a maximum of 3 min. Each mouse was tested in three separate trials, and the average time was calculated.

### 2.5 Olfactory behaviour tests

Olfactory behaviour was tested as previously described,^25^ with minor modifications. Notably, experiments were performed in an open field. Four vessels (3 × 3 × 3 cm) were placed in one corner of the test box (72 × 72 × 30 cm). The test was divided into four stages, that is acclimation, habituation, exposure to the vehicle and exposure to a test substance in the vehicle. Before testing, mice were individually acclimated for 15 min.

For the surface pellet test, the test substance was placed in one corner (vehicle and test substance), whereas the other three corners contained the vehicle only. Each mouse underwent three consecutive trials where they were presented with Kellogg's Coco Pops cereal on the surface of 3 cm‐thick clean bedding. The period in which the mouse remained in each corner was recorded until the pellet was found in 5 min.

For the buried pellet test, mice were individually housed, and their diet was restricted until their body weight decreased to 90% of the starting value. A clean cage was covered with 3 cm of clean bedding for the test, with Coco Pops cereal, female mouse urine or the vehicle buried 0.5 cm below the surface. The mouse was placed in the cage, and the time it took to dig up the food was recorded to a maximum of 5 min. The preference index was established as the time spent in the corner with the test substance minus the average time spent in the other corners.

### 2.6 Caspase‐3 activity assay

Tissue samples (5 mg) were homogenized and incubated in 120 μl lysis buffer on ice for 10 min, followed by centrifugation at 12,000 × *g* at 4°C for 10 min. The soluble fraction was transferred to a 1.5‐ml centrifuge tube, and protein concentration was determined using the Bradford assay kit (GenMed Scientifics, Shanghai, China). Caspase‐3 activity was assessed using the Caspase‐3 Colorimetric Assay kit (Applygen Technologies) as per the manufacturer's instructions.

### 2.7 Isolation of membrane and cytoplasmic fractions

Membrane and cytoplasmic fractions were extracted using the Mem‐PER Plus Membrane Protein Extraction kit (Thermo Fisher Scientific) based on the manufacturer's instructions.

### 2.8 Primary olfactory bulb neuron cultures

As previously described, primary neurons from olfactory bulbs of newborn mice (P0–P1) were dissociated.^26^ Neurons were cultured in poly‐l‐lysine‐coated 6‐ or 24‐well plates or glass‐bottomed culture dishes (NEST, Wuxi, China) containing Neurobasal‐A medium supplemented with B27, L‐glutamine and 100‐units/ml penicillin/streptomycin for 5 days before being infected with LV plasmids. The cells grew for additional 7 days with periodic changes in the medium. Some cultures were selected to determine the purity of neurons by calculating the percentage of NSE‐positive versus total cells. Above 90% of cells were neurons under the present culture conditions.

### 2.9 Immunohistochemistry and confocal microscopy

Mice were anaesthetised using 8% chloral hydrate and perfused with physiological saline followed by 4% paraformaldehyde. The OB and OE were removed and cryo‐protected with 20% sucrose before sectioning into 20‐μm sections. After blocking with 5% goat serum, the cells were incubated overnight at 4°C with primary antibodies against α‐syn, TH, TRPC3, caspase‐3 and Omp, followed by a 1‐h incubation at room temperature with Alexa Fluor 448/594/647‐conjugated secondary antibodies (1:500; Invitrogen). Cell nuclei were counterstained with DAPI (Sigma) for 5 min. Sections were mounted on slides, coverslipped and visualized under a confocal microscope (Leica).

Primary OB neurons seeded on coverslips were fixed with 4% paraformaldehyde for 20 min, then permeabilized using 0.3% Triton X‐100 for 20 min. Neurons were labelled with similar primary and secondary antibodies used for tissue sections and observed under confocal microscopy.

### 2.10 Western blot analysis

Primary neurons and tissue samples were homogenized on ice in radioimmunoprecipitation (RIPA) lysis buffer (Sigma) containing phosphatase and protease inhibitors. Homogenates were centrifuged at 12,000 × *g* for 15 min; the supernatant was obtained and 30 μg of protein were separated by 15% sodium dodecyl sulphate‐polyacrylamide gel electrophoresis (SDS‐PAGE) then transferred to polyvinylidene difluoride membranes (Millipore). After blocking with 5% milk for 1 h, membranes were incubated overnight at 4°C with primary antibodies against the following proteins: human α‐syn (3D5), mouse α‐syn, Omp, TH, caspase‐3, β‐actin, TRPC3, TRPC6, calnexin, GAPDH and p‐Tyr. Protein bands were visualized by enhanced chemiluminescence, and signal intensity was measured by densitometry using a Versadoc XL imaging system (Bio‐Rad).

### 2.11 High‐performance liquid chromatography (HPLC)

Fresh‐frozen tissue samples were homogenized on ice in 0.3 M perchloric acid to measure dopamine (DA) levels and its metabolites in tissue. The homogenate was centrifuged at 15,000 × *g* at 4°C for 15 min. The supernatant was diluted at 1:2 in the mobile phase, and phosphoric acid was added to adjust the pH of the mixture. DA, 3,4‐dihydroxyphenylacetic acid (DOPAC) and homovanillic acid (HVA) concentrations were measured by HPLC with colorimetric, electrochemical detection and expressed as ng/mg tissue. Dopaminergic neuron activity was represented as the turnover rate for DA [(DOPAC+HVA)/DA].

### 2.12 Immunoprecipitation (IP)

Protein enrichment was examined by IP as previously described.^20^ Briefly, the OB and OE from *Thy1‐α‐syn* or WT mice were lysed in Tris‐NaCl‐EDTA buffer comprising 50 mm Tris–HCl (pH 7.4), 100 mm NaCl, 0.1 mm EDTA and centrifuged at 12,000 × *g*. The supernatant containing 100 μg protein was incubated with an anti‐TRPC3 antibody (3 μg) overnight at 4°C under constant rotation. Subsequently, the protein‐antibody mixture was incubated at 4°C for 1 h under constant rotation with protein G‐sepharose beads washed with IP buffer. The beads were collected by centrifugation, and nonspecifically bound proteins were removed by washing with IP buffer. Washed beads were re‐suspended in SDS‐PAGE loading buffer (30 μl/tube) and heated at 95°C for 5 min. The beads were removed by centrifugation, and the supernatant was analysed by Western blotting.

### 2.13 Intracellular Ca^2+^ imaging

Changes in intracellular free Ca^2+^ concentration ([Ca^2+^]i) were determined using the fluorescent Ca^2+^ indicator Fluo‐4 acetoxymethyl (AM) ester (1 μM; Dojindo Laboratories) as previously described.^27^ Primary olfactory bulb neurons were cultured in glass‐bottomed culture dishes for 12 days. To prevent interference from endogenous Ca^2+^ stores in the endoplasmic reticulum (ER) and mitochondria, neurons were pretreated with cyclosporine A (CsA, 5 μM; Cell Signalling Technology) to block the mitochondrial permeability transition pore; CGP37157 (10 μM; Abcam) to block the mitochondrial sodium/calcium exchanger; flufenamic acid (FFA, 40 μM; Sigma) to block ER Ca^2+^ flux; and the inositol 1,4,5‐trisphosphate receptor inhibitor xestospongin C (Xes, 1 mM; Sigma).

After washing with Hank's balanced salt solution (HBSS) without Ca^2+^ or Mg^2+^, neurons were incubated in HBSS containing Fluo 4‐AM at 37°C for 45 min in a humidified incubator of 5% CO_2_. To remove extracellular Fluo 4‐AM, neurons were washed twice in HBSS (without Ca^2+^ or Mg^2+^) and then incubated in HBSS (with Ca^2+^) at 37°C, away from light for 20 min. To measure [Ca^2+^]i, the fluorescence was monitored in randomly selected cells using a Live Cell Imaging System (Leica AF7000) with excitation and emission wavelengths of 495 and 518 nm, respectively. When the baseline fluorescent signal had been stable for 180 s, 1‐oleoyl‐2‐acetyl‐sn‐glycerol (OAG, 10 μM; Sigma) or hyperforin (10 μM; Sigma) was added, and cells were recorded for an additional 15 min. Images were obtained at a 1 frame/10 s and analysed using the Leica software. Peak values of [Ca^2+^]i were quantified by calculating the F_*t*_/F_0_ ratio, where F_*t*_ represents the fluorescence intensity of the Ca^2+^ indicator at time *t* (in seconds) after adding OAG or hyperforin, and F_0_ is the fluorescence intensity of the Ca^2+^ indicator before adding OAG or hyperforin. Neurons from *α‐syn* Tg and WT mice were divided into different groups, some of which were pretreated with ethyl‐1‐(4‐[2,3,3‐trichloroacrylamide] phenyl)‐5‐(trifluoromethyl)‐1H‐pyrazole‐4‐carboxylate (Pyr3, 10 μM; Sigma) or 1‐[2‐[3‐(4‐Methoxyphenyl) propoxy]‐2‐(4‐methoxyphenyl) ethyl]‐1H‐imidazole hydrochloride (SKF96365, 10 μM; Sigma). OAG or hyperforin was administered after 5 min incubation in the presence of inhibitors.

### 2.14 Evaluation of cell viability rates

Cell viability rates were evaluated with calcein acetoxymethyl ester (calcein‐AM, Dojindo Laboratories) and propidium iodide (PI, Sigma) staining. Briefly, cultures were incubated at 37°C for 20 min with a solution of calcein‐AM (2 μM) and PI (4.5 μg/ml). Living cells (green cytoplasmic fluorescence) and dead cells (red nuclei) in 10 visual fields per well of a 24‐well plate were observed and analysed using the GE IN Cell Analyser 2000 High‐Content Cellular Analysis System (GE Healthcare Biosciences) to evaluate the ratio of cell viability presented as a ratio of calcein‐AM positive cells/PI‐positive cells. We measured four wells for each group.

### 2.15 Determination of apoptotic cell death rates

Apoptotic cell death rates were established using the transferase‐mediated deoxyuridine triphosphate (dUTP)‐digoxigenin nick‐end labelling (TUNEL) assay.^20^ Briefly, neurons were fixed using 4% paraformaldehyde and processed using the In Situ Cell Death Detection Kit (Roche) as per the manufacturer's instructions. Cell nuclei were stained with Hoechst 33342 and observed under a fluorescent microscope. Data were expressed as the ratio of the TUNEL‐positive versus total neurons in ten random fields per well of a 24‐well plate. Four wells were measured for each group.

### 2.16 Statistical analysis

Data were expressed as mean ± SD. Statistical analyses were performed using the GraphPad Prism v.6.0 software (GraphPad). Differences between groups were assessed with Student's *t*‐test and by two‐way analysis of variance followed by Tukey's multiple comparisons test. *p* < 0.05 was considered statistically significant.

## 3 RESULTS

### 3.1 Olfactory function was impaired before motor deficits in *α‐syn* Tg mice

**FIGURE 1:**
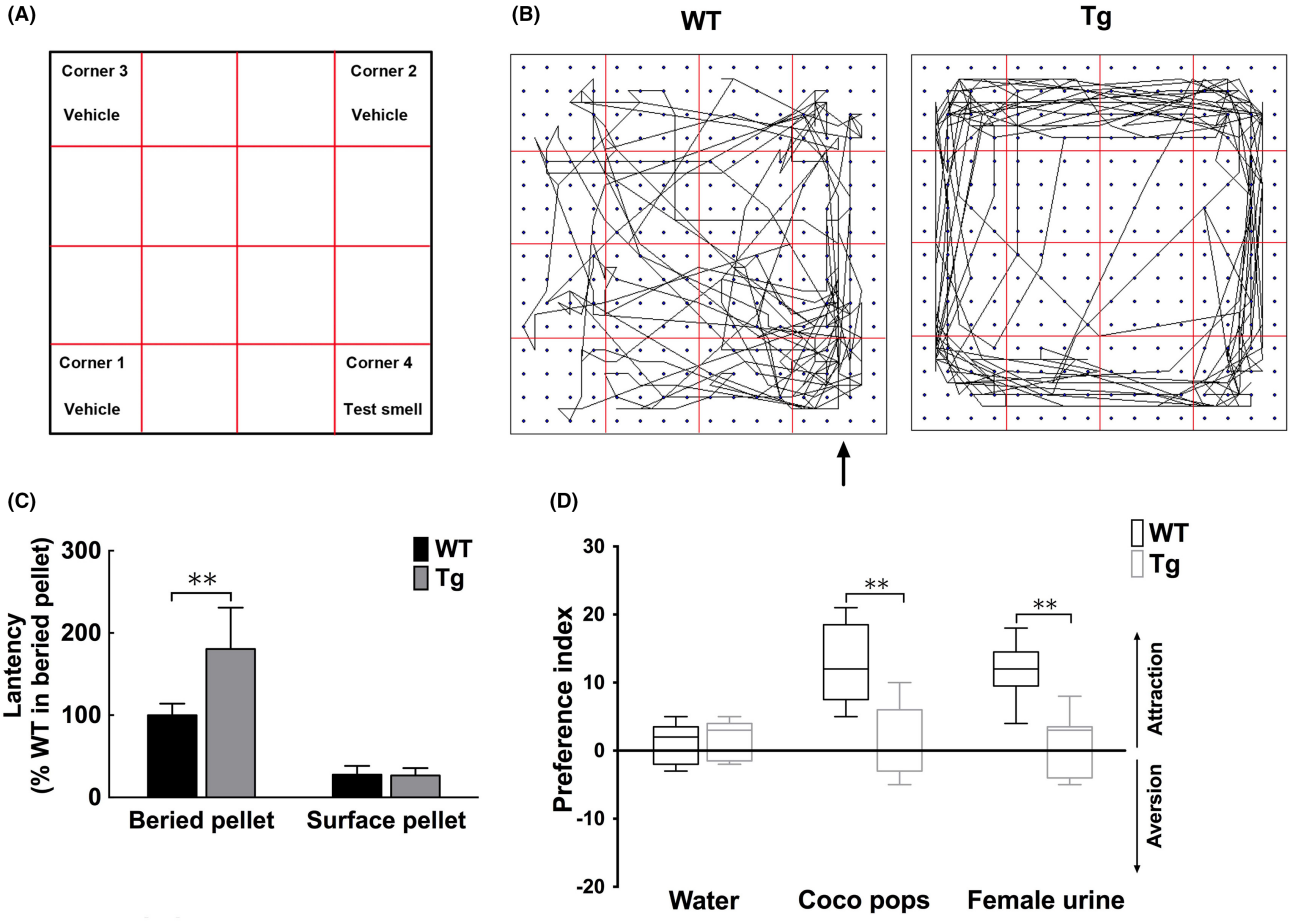
Olfactory deficits were noted in 6‐month α‐syn Tg mice. (A) Schematic presentation of the open field. (B) Representative locomotor traces of WT and α‐syn Tg mice in the open field during a 5‐min exposure to test substance (indicated by the arrow) or vehicle. (C) Latency to uncover the pellet in the buried pellet test (two‐way analysis of variance; *n* = 10). (D) Preference index values for α‐syn Tg mice (white columns) and WT littermates (black columns). Coco Pops cereal and female mice urine were used as olfactory cues, with water as the control. Preference index = time spent in corner 4 ‐ average time spent in the other corners. Data are shown as mean ± SD. Tg, transgenic; WT, wild‐type

Previous studies indicate that olfactory function is impaired in *α‐syn* Tg mice.^7^ To verify these results, we utilizing surface and buried test to evaluated the olfactory function of the 6‐month‐old male *mThy1‐α‐syn* Tg mice. The softmaze system was used to record the trajectory of mice (Figure 1A,B).In contrast with the WT controls, *α‐syn* Tg mice took a longer time to locate the buried pellet (Figure 1C) despite the two groups showing similar latencies to identify the surface pellets. In addition, in response to freshwater, all mice displayed comparable olfactory behaviour. In contrast, Coco Pops cereal and female mouse urine were more attractive to WT than to *α‐syn* Tg mice (Figure 1D). These results indicate that the longer time by *α‐syn* Tg mice was attributed to functional olfactory reduction but not inattentiveness or motor impairment. In support of this conjecture, motor performance was similar between *α‐syn* Tg and WT mice in the rotarod test (Figure S1A). Using immunohistochemistry and Western blots, we detected the level of tyrosine hydroxylase (TH), the rate‐limiting enzyme in the synthesis process of dopamine substantia nigra (SN) and striatum (STR), to evaluate the loss of dopaminergic neurons or fibres. There was no significant difference in the TH level between WT or *α‐syn* Tg mice tissues (Figure S1B–D). Also, we measured the contents of DA and its metabolites (DOPAC and HVA) by HPLC. Consequently, we found no differences in DA, DOAPC or HVA levels in the brain regions mentioned above nor in the (DOPAC + HVA)/DA ratio between *α‐syn* Tg mice and their WT littermates (Figure S1E,F). These findings indicate that olfactory function was impaired in 6‐month *α‐syn*‐overexpressing mice and dysosmia occurred earlier than motor deficits.

### 3.2 Dopaminergic neurons were not implicated in olfactory dysfunction of *α‐syn* Tg mice

Studies have shown that reduced DA synthesis and release potentially impair olfactory function.^28^, ^29^ Therefore, we assessed dopaminergic neurons in the OB and OE of *α‐syn* Tg mice. Human WT *α‐syn* was detected by Western blotting using the 3D5 monoclonal antibody, recognizing a specific sequence of *hα‐syn* ^23^. *Hα*‐*syn* was detected in the OB, OE of *α‐syn* Tg but not in WT mice (Figure S2A). In contrast, both *α‐syn* Tg and WT mice expressed similar levels of mouse *α‐syn* in these brain regions (Figure S2A,B). Considering that previous studies have shown that α‐syn overexpression inhibits TH expression,^30^ we speculated that TH downregulation reduces DA synthesis and its subsequent release from dopaminergic neurons in the OB and OE, which might impair olfactory function. Unexpectedly, we found no reduction in TH‐positive cells (Figure S2D) or TH expression levels in the OB, OE (Figure S2A,C) in *α‐syn* Tg compared with WT mice. No differences were found in DA, DOAPC or HVA levels nor in the (DOPAC + HVA)/DA ratio between OB or OE of Tg mice and their WT littermates (Figure S2E,F). These findings reveal that the olfactory dysfunction in *α‐syn* Tg mice was not attributed to the impairment of dopaminergic neurons.

### 3.3 Apoptosis induction occurred in the OB and OE of *α‐syn* Tg mice

**FIGURE 2:**
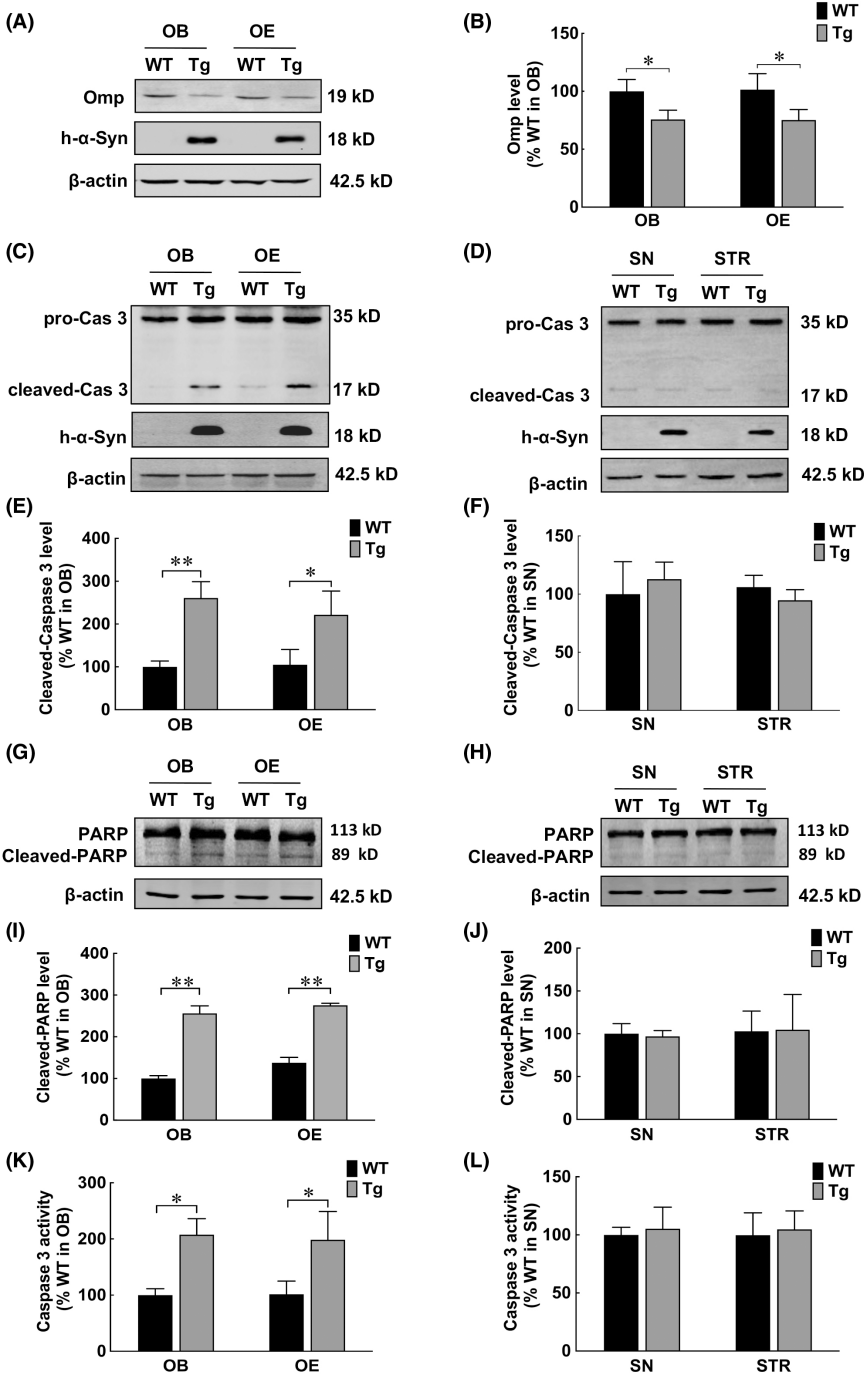
Caspase‐3 was activated in the olfactory systems of α‐syn Tg mice. (A, B) Western blot and quantitative analyses of Omp in OB and OE. (C, E) Western blot and quantitative analyses of cleaved caspase‐3 levels in OB and OE of α‐syn Tg and WT mice. (D, F) Western blot and quantitative analyses of cleaved caspase‐3 levels in SN and STR. (G, I) Western blot and quantitative analyses of cleaved PARP levels in OB and OE of α‐syn Tg and WT mice. (H, J) Western blot and quantitative analyses of cleaved PARP levels in SN and STR. (K) Caspase‐3 activities in OB and OE. (L) Caspase‐3 activities in SN and STR. Data are expressed as mean ± SD (two‐way analysis of variance). *p < 0.05, **p < 0.01 (*n* = 3). OB, olfactory bulb; OE, olfactory epithelium; SN, substantia nigra; STR, striatum; Tg, transgenic; WT, wild‐type

The mature olfactory neuron marker, Omp was measured to investigate the mechanism of α‐syn overexpression‐induced olfactory dysfunction. Western blot results revealed downregulated Omp levels in both OB and OE of *α‐syn* Tg mice (Figure 2A,B), indicating that mature olfactory neurons were reduced in the olfactory system of *α‐syn* Tg mice. Thus, we speculated increased apoptosis level was involved in resulting in olfactory impairment. To test this hypothesis, apoptosis levels were evaluated via cleaved caspase‐3, caspase‐3 activities and cleaved PARP, the most critical substrate for caspase3. Although no differences were observed in the SN or STR (Figure 2D,F,H,J,L), about a 40% increase in cleaved Casp3 in OB neurons (Figure S3) and potentiated PARP and caspase3 activities were found in the OB and OE of *α‐syn* Tg mice compared with that of WT mice (Figure 2C,E,G,I,K). These findings indicate that the olfactory system is more susceptible to apoptosis upon α‐syn overexpression than the nigrostriatal system.

### 3.4 TRPC3 activated in the OB and OE of *α‐syn* Tg mice

**FIGURE 3:**
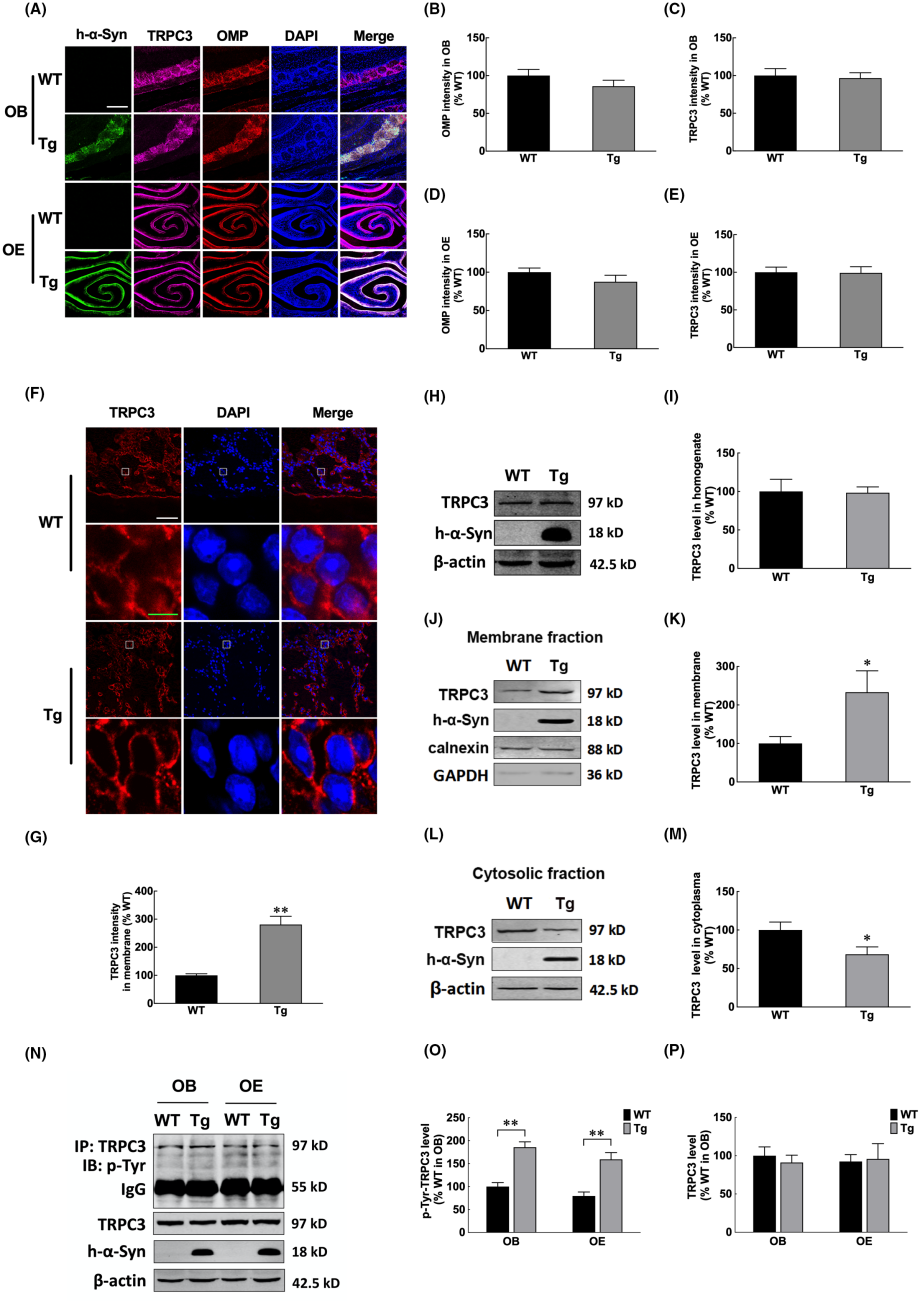
TRPC3 was activated in OB and OE of α‐syn Tg mice. (A–E) Photomicrographs and statistical analyses of TRPC3 and Omp levels in OB and OE of α‐syn Tg and WT mice. Scale bar = 500 μm. (F) Photomicrographs and statistical analyses of TRPC3 levels in OB of α‐syn Tg and WT mice. Boxed areas are higher magnification. Scale bar (white) = 50 μm; scale bar (green) = 10 μm. (H, I) Western blotting and quantitative analyses of TRPC3 protein levels in OB. (J–M) Western blotting and quantitative analyses of TRPC3 protein levels in the membrane (J, K) or cytosolic (L, M) fractions of the OB of α‐syn Tg and WT mice. (N–P) Evaluation of TRPC3 protein levels by IP; Precipitated amounts of p‐Tyr (reflecting TRPC3 activation) were estimated by western blotting. Data in IP are expressed as mean ± SD (two‐way analysis of variance). **p < 0.01 (*n* = 3). The other data are expressed as mean ± SD (Student's *t*‐test). *p < 0.05, **p < 0.01 vs. WT (*n* = 3). OB, olfactory bulb; OE, olfactory epithelium; Tg, transgenic; WT, wild‐type

Ca^2+^ has a biphasic effect on cell growth, that is a modest increase in Ca^2+^ influx stimulates cell proliferation, whereas a rapid influx causes cell death.^31^ TRPC channels belong to a Ca^2+^‐permeable, non‐selective cation channel superfamily involved in sensory transduction and cell growth. Additionally, since α‐syn overexpression activates Src tyrosine kinase,^20^ an upstream modulator of TRPC3, we speculated that TRPC3 expression and activity would be disrupted in the olfactory system of *α‐syn* Tg mice. As identified by immunocytochemistry, TRPC3‐positive cells were localized in the olfactory nerve and glomerular layers of OB as well as in the outer layer towards the nasal cavity of the OE in both the *α‐syn* Tg and WT mice (Figure 3A–E). Western blot analysis revealed similar levels of TRPC3 between the two groups (Figure 3H,I). Nonetheless, TRPC3 levels were higher in the OB membrane and lower in the cytosolic fractions of *α‐syn* Tg mice than WT mice, as identified by immunocytochemistry (Figure 3F,G) and Western blotting (Figure 3J–M); this indicates that *α‐syn* overexpression causes membrane recruitment of TRPC3 protein. Accordingly, the phosphorylated TRPC3, an activated form of channel,^32^ also increased in the OB of *α‐syn* Tg mice compared with the WT controls (Figure 3N–P).

Since Src tyrosine kinase regulatesTRPC6, a protein closely related to TRPC3,^33^ we examined its expression levels and activity in *α‐syn* Tg mice. The results showed no differences in TRPC6 levels between *α‐syn* Tg and WT mice in either whole‐cell homogenates (Figure S4A,B), membrane fractions (Figure S4C,D) or cytosolic fractions (Figure S4E,F). Moreover, the levels of activated phosphorylated TRPC6^34^ in the OB were not significantly different between *α‐syn* Tg mice and WT littermates (Figure S3G). The above findings indicate that TRPC3, but not TRPC6, is upregulated in the OB and OE of *α‐syn* Tg mice.

### 3.5 *α‐Syn* overexpression improved TRPC3‐induced Ca^2+^ influx and apoptosis in primary olfactory neurons

TRPC3 is closely linked to intracellular Ca^2+^ homeostasis; its overexpression and activation in the neuronal membrane potentially trigger the entry of excess extracellular Ca^2+^, perturbing intracellular Ca^2+^ levels, thereby causing apoptosis. To establish whether *α‐syn* overexpression affects this process, we examined *α‐syn* and TRPC3 expression in primary cultures of olfactory neurons isolated from *α‐syn* Tg and WT mice. TRPC3 protein was expressed at comparable levels in the two groups (Figure S5A,B). Nevertheless, the membrane localization of TRPC3 was higher in neurons from *α‐syn* Tg than in WT mice (Figure S5C), consistent with observations in tissue sections.

**FIGURE 4:**
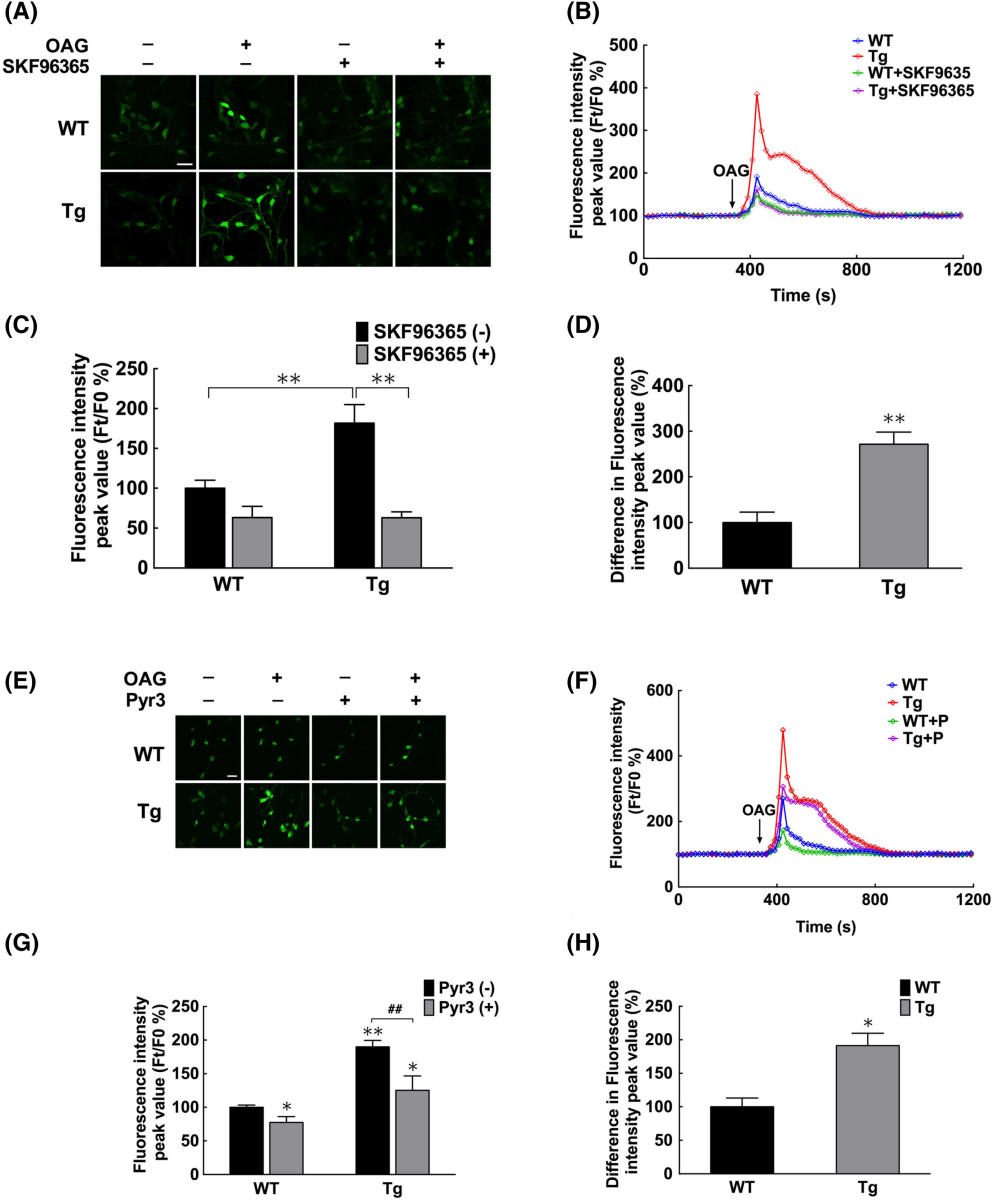
TRPC3 increased Ca^2+^ influx in primary olfactory neurons. (A) Photomicrographs showing fluorescence intensities upon treatment with the TRPC3 agonist (OAG) and/or TRPC blocker (SKF96365). Scale bar = 20 μm. (B) Changes in Ca^2+^ influx as determined by the fluorescence intensity ratio (F_*t*_/F_0_). F_*t*_, fluorescence intensity of the indicator at *t* seconds after treatment with OAG; F_0_, fluorescence intensity of the indicator at 0 seconds. (C) Peak fluorescence intensity values before and after OAG treatment. Peak Ca^2+^ influx values were quantified by evaluating F_*t*_/F_0_ after OAG addition (two‐way analysis of variance; **p < 0.01, *n* = 3). (D) Effects of TRPC on Ca^2+^ influx, as determined by differences in peak fluorescence intensity values between the groups with or without SKF96365 treatment (Student's *t*‐test; **p < 0.01 vs. WT, *n* = 3). (E) Photomicrographs showing fluorescence intensities upon treatment with the TRPC3 agonist (OAG) and/or the selective TRPC3 inhibitor (Pyr3). Scale bar = 20 μm. (F) Changes in Ca^2+^ influx as determined by the fluorescence intensity ratio (F_*t*_/F_0_). (G) Peak fluorescence intensity values before and after OAG treatment. Peak Ca^2+^ influx values were quantified by calculating F_*t*_/F_0_ after the addition of OAG (Two‐way analysis of variance, *p < 0.05, **p < 0.01 vs. WT without Pyr3/Lentivirus treatment; ^##^ *p* < 0.01; *n* = 3). (H) Effects of TRPC3 on Ca^2+^ influx, as determined by differences in peak values of fluorescence intensity between groups with or without Pyr3 treatment (Student's *t*‐test, *p < 0.05 vs. WT; *n* = 3). Tg, transgenic; WT, wild‐type

Subsequently, changes in [Ca^2+^]i were measured in response to TRPC3 activation. Neurons were pretreated with Ca^2+^ channel blockers including CsA, CGP37157, FFA and Xes to eliminate interference from Ca^2+^ released from mitochondria and ER. After treatment with OAG, a TRPC3 activator, [Ca^2+^]i markedly increased, an effect that was ameliorated by TRPC inhibitor, SKF96365 (Figure 4A–D) or *Pyr3*, a TRPC3 selective inhibitor (Figure 4E–H). Furthermore, the [Ca^2+^]i increase was more significant in neurons isolated from *α‐syn* Tg than those isolated from WT mice (Figure 4E–H).

**FIGURE 5:**
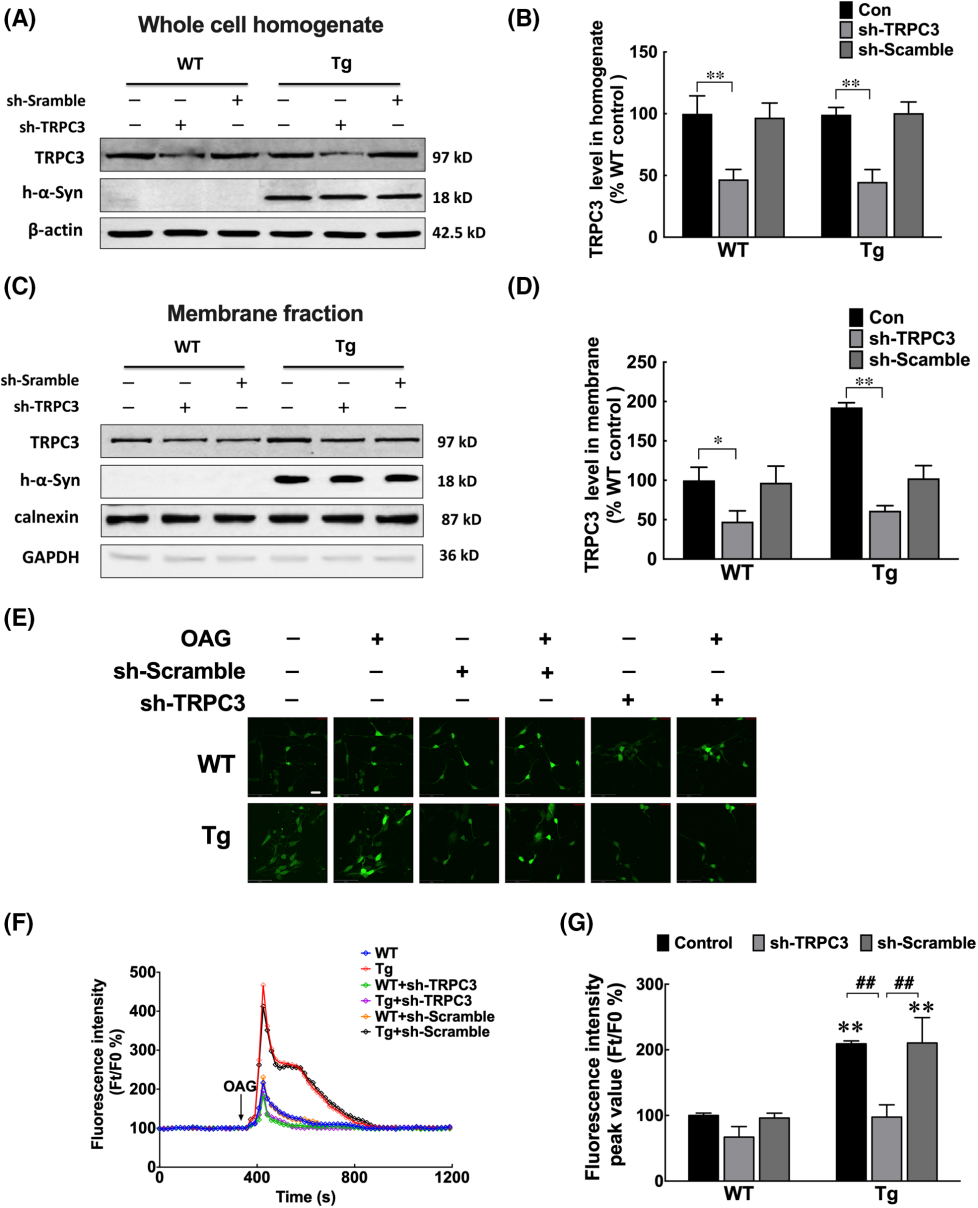
Knockdown of *TRPC3* inhibited the increase in Ca^2+^ influx in primary olfactory neurons. (A–D) Western blotting and quantitative analyses of TRPC3 levels in whole cell homogenates (A, B) or membrane fractions (C, D) of primary olfactory neurons of α‐syn Tg and WT mice infected with LV‐sh‐*TRPC3* or LV‐sh‐Scramble for 7 days. (E) Photomicrographs showing fluorescence intensities upon treatment with OAG and/or *TRPC3* knockdown. Scale bar = 20 μm. (F) Changes in Ca^2+^ influx as determined by the fluorescence intensity ratio (F_*t*_/F_0_). (G) Peak fluorescence intensity values before and after OAG treatment. Peak Ca^2+^ influx values were quantified by calculating F_*t*_/F_0_ after OAG addition. (A–D) Data are expressed as mean ± SD (two‐way analysis of variance). *p < 0.05, **p < 0.01 (*n* = 3). (G) Two‐way analysis of variance, *p < 0.05, *p < 0.05, **p < 0.01 vs. WT without Pyr3/Lentivirus treatment; ^##^ *p* < 0.01 (*n* = 3). Tg, transgenic; WT, wild‐type

To further clarify the effect of *α‐syn*‐induced TRPC3 activation on [Ca^2+^] i, we evaluated the changes in [Ca^2+^]i after treating neurons with shRNA to knock down *TRPC3* expression (Figure 5A–D). The results showed that the improved [Ca^2+^]i in neurons from *α‐syn* Tg mice were significantly reduced after administering shRNA (Figure 5E–G). To further exclude the role of TRPC6 for the improved [Ca^2+^]i, hyperforin (10 μM), a TRPC6 specific agonist^35^, ^36^ was added to stimulate the neurons. No further increase of [Ca^2+^]i was noted in neurons from *α‐syn* Tg mice than those from WT littermates (Figure S6A–C).

**FIGURE 6:**
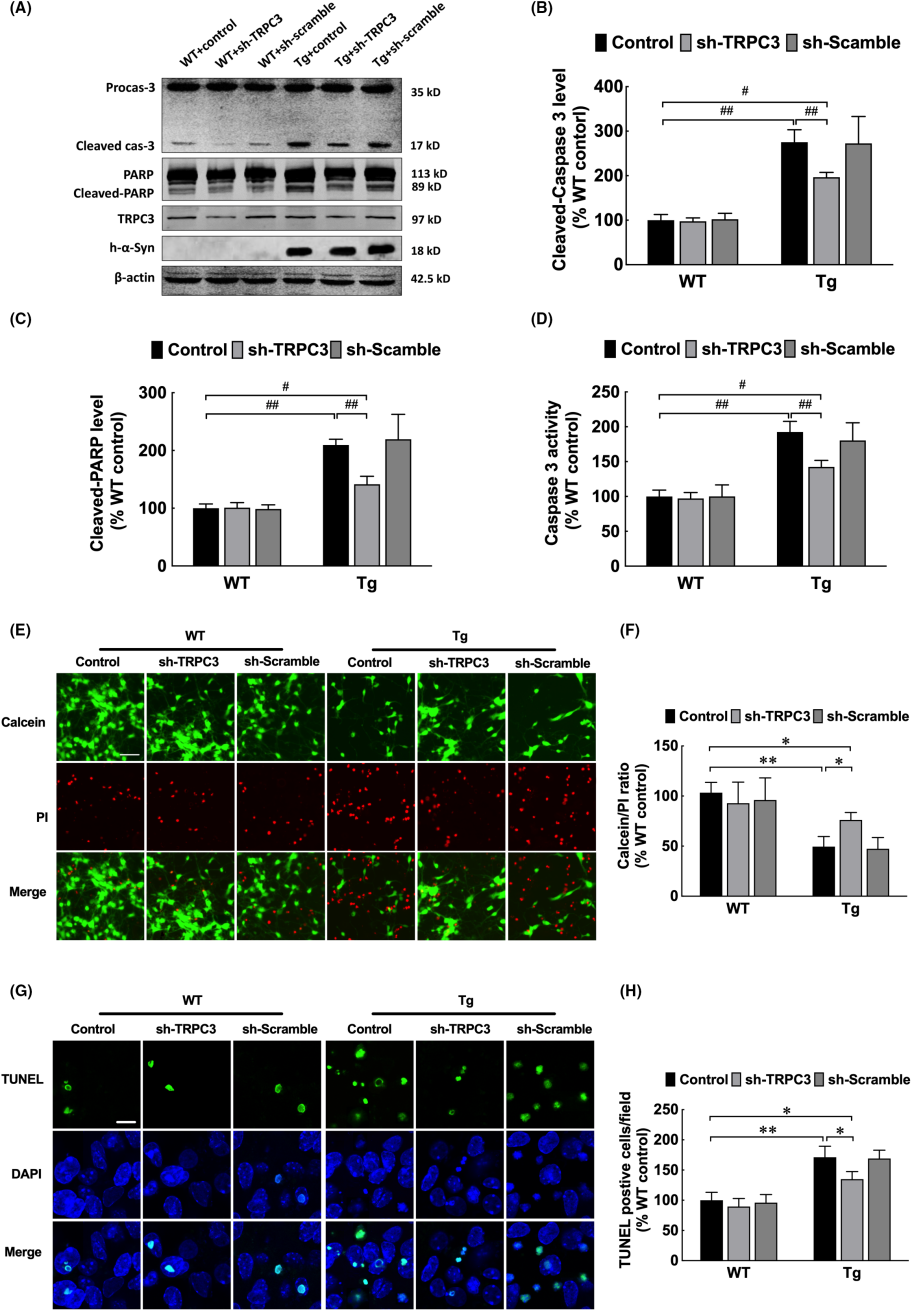
*TRPC3* knockdown suppressed α‐syn overexpression‐induced apoptosis of primary olfactory neurons. (A–C) Western blotting and quantitative analyses of cleaved caspase‐3 and cleaved PARP levels in primary olfactory neurons, both of which were reduced by *TRPC3* knockdown in Tg mice. (D) Quantitative analyses of caspase‐3 activities. (E, F) Photomicrographs and statistical analyses of primary olfactory neurons stained with calcein‐AM/PI. Scale bar = 50 μm. (G, H) Photomicrographs and statistical analyses of primary olfactory neurons with TUNEL staining. Data are expressed as mean ± SD (two‐way analysis of variance). (B, C) ^#^ *p* < 0.05, ^##^ *p* < 0.01 (*n* = 3). (E, G) *p < 0.05, **p < 0.01 (*n* = 4). Tg, transgenic; WT, wild‐type

The activated caspase‐3 expression after TRPC3 activation was evaluated to establish whether apoptosis is induced by increasing intracellular Ca^2+^ levels. Cleaved caspase‐3 levels, cleaved PARP levels (Figure 6A–C) and caspase‐3 activities (Figure 6D) increased in olfactory neurons from *α‐syn* Tg mice. These effects were reversed by knocking down *TRPC3* expression (Figure 6A–D). Based on the improved caspase‐3 activity, the apoptosis rate increased by calcein‐AM/PI staining (Figure 6E,F) and TUNEL assay (Figure 6G,H); similarly, these increased apoptotic levels in primary OB neurons isolated from *α‐syn* Tg mice were reversed after shRNA‐mediated knockdown of *TRPC3*.

## 4 DISCUSSION

Previous studies indicate that olfactory function is impaired in *α‐syn* Tg mice.^7^, ^37^, ^38^, ^39^ In line with these findings, we found that *α‐syn* overexpressed mice developed dysosmia. In addition to deficits in olfactory function, we observed a reduction in the mature olfactory neuron marker levels in the OB and OE of the *α‐syn* Tg mice. Additionally, we found increased apoptotic levels in the OB and OE of the *α‐syn* Tg mice. Our findings suggest that α‐syn overexpression decreased the number of mature olfactory neurons by inducing apoptosis, further contributing to olfactory dysfunction.

Several studies indicate that TRPC3 is implicated in regulating cell function and fate by mediating Ca^2+^ signalling.^40^ Our previous work revealed that TRPC3 is involved in α‐syn accumulation in the brains of mice and monkeys.^21^ We also found that α‐syn overexpression could activate Src tyrosine kinase, an upstream modulator of TRPC3.^20^ Since the role of TRPC3 in α‐syn overexpression‐induced olfactory dysfunction remains unclear, we hypothesized that TRPC3 modulates this pathological process. Furthermore, we discovered TPRC3 activation and translocation on the plasma membrane in the OB and OE of α‐syn Tg mice. In primary olfactory neurons, α‐syn overexpression increased the levels of TRPC3 on membrane fraction, indicating that α‐syn overexpression triggers TRPC3 activation. In these neurons, TRPC3 activation significantly increased intracellular Ca^2+^ concentration, whereas caspase‐3 activity and apoptosis rate increased concomitantly. Nevertheless, knocking down TRPC3 expression abrogated caspase‐3 activation and cell death and caused a TRPC3‐mediated intracellular Ca^2+^ increase. Multiple evidence have proved the relationship between increased intracellular Ca^2+^ and induction of caspase activity. For example, A Gennari et al. showed that the apoptotic pathway followed by organotin compounds starts with an increase of intracellular Ca^2+^, then continues with release of ROS and cytochrome c from mitochondria, activation of caspases, and finally results in DNA fragmentation.^41^ Cypermethrin was found to increase apoptosis rate of TM4 cells significantly and with a significant increase in intracellular Ca^2+^ concentration.^42^ Based on our experimental results and the above analysis, we believe that TRPC3‐mediated Ca^2+^ influx caused significant α‐syn overexpression‐mediated apoptosis.

Since Src tyrosine kinase regulates TRPC6 protein,^33^ we evaluated its expression and activity. The results showed no differences in membrane‐bound or phosphorylated TRPC6 levels in *α‐syn* Tg and WT mice. In line with these findings, TRPC6 specific agonist did not induce additional [Ca^2+^]i increase in primary olfactory neurons of *α‐syn* Tg mice compared with WT mice. Collectively, our findings suggest that α‐syn overexpression activated TRPC3, but not TRPC6, further increasing intracellular Ca^2+^ concentration and inducing apoptosis.

**FIGURE 7:**
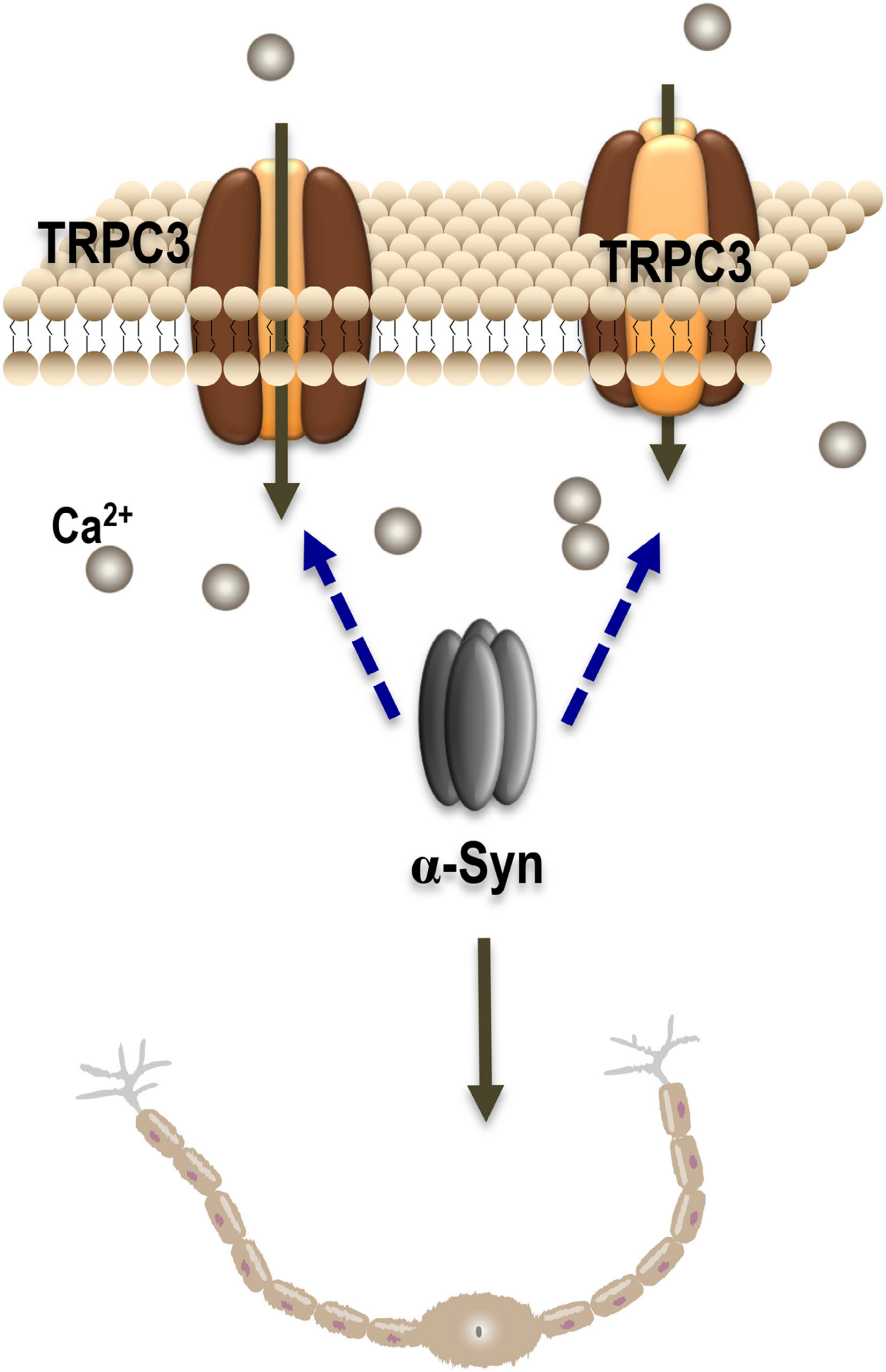
TRPC3 activation‐induced calcium overload impaired neurogenesis in α‐syn overexpressing mice. In α‐syn transgenic mice, α‐syn overexpression induced TPRC3 membrane recruitment and activation (the specific mechanism should be investigated), resulting in abnormal elevations of intracellular Ca^2+^ levels. Calcium overload impaired olfactory sensory neurons by increasing the apoptosis rates

Olfactory dysfunction is an early symptom of PD caused by abnormal changes of α‐syn in the OB and OE. This work indicates that α‐syn overexpression induces abnormal TRPC3 activation, imbalance of calcium homeostasis and apoptosis (Figure 7), which all promote dysosmia in *α‐syn* Tg mice. Our findings provide novel insights into the mechanistic basis for olfactory dysfunction in PD patients. We also highlight that TRPC3 is a potential target in this pathological process.

## AUTHOR CONTRIBUTIONS

**Min Chen:** Conceptualization (lead); data curation (equal); formal analysis (lead); methodology (equal); writing – original draft (lead). **Jia Liu:** Data curation (equal); formal analysis (lead); investigation (equal); methodology (equal); writing – original draft (equal). **Hanjiang Luo:** Data curation (equal); formal analysis (equal); investigation (equal); methodology (equal); writing – original draft (equal). **Ge Gao:** Methodology (equal). **Duan Chunli:** Methodology (equal). **Hui Yang:** Funding acquisition (lead); project administration (lead); writing – review and editing (lead).

## CONFLICT OF INTEREST

The authors declare no conflicts of interest.

## Supporting information


Figure S1

Figure S2

Figure S3

Figure S4

Figure S5

Figure S6

## Data Availability

The data that support the findings of this study are available from the corresponding author upon reasonable request.
