# Supplementary figures and images for: Increase in membrane surface expression and phosphorylation of TRPC3 related to olfactory dysfunction in α‐synuclein transgenic mice
Source: J Cell Mol Med. 2022 Aug 27;26(19):5008–20. doi: 10.1111/jcmm.17524 (PMC9549507; doi:10.1111/jcmm.17524)

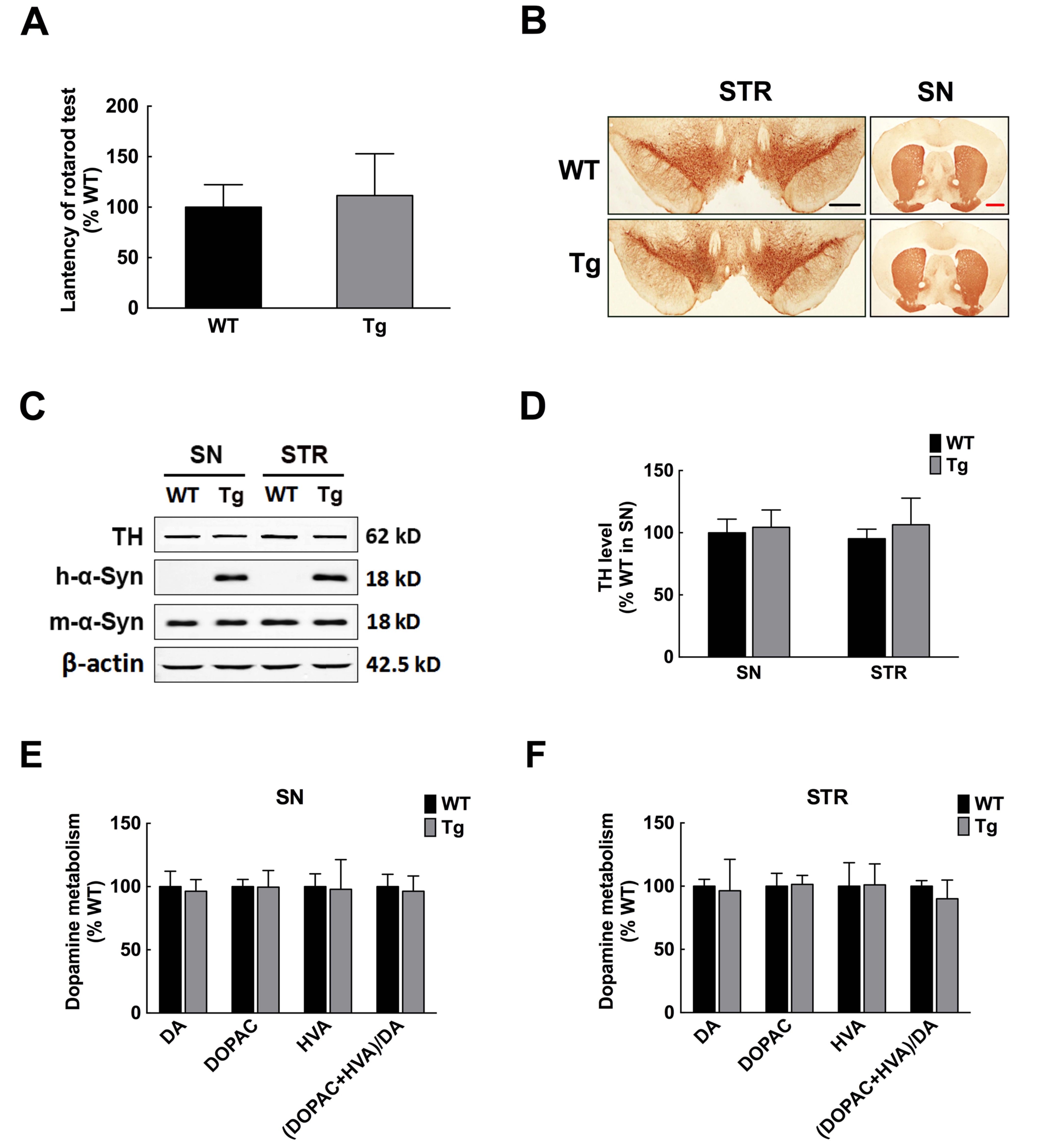

Supplement: Supplementary file 1 — Figure S1 [file JCMM-26-5008-s006.jpg]

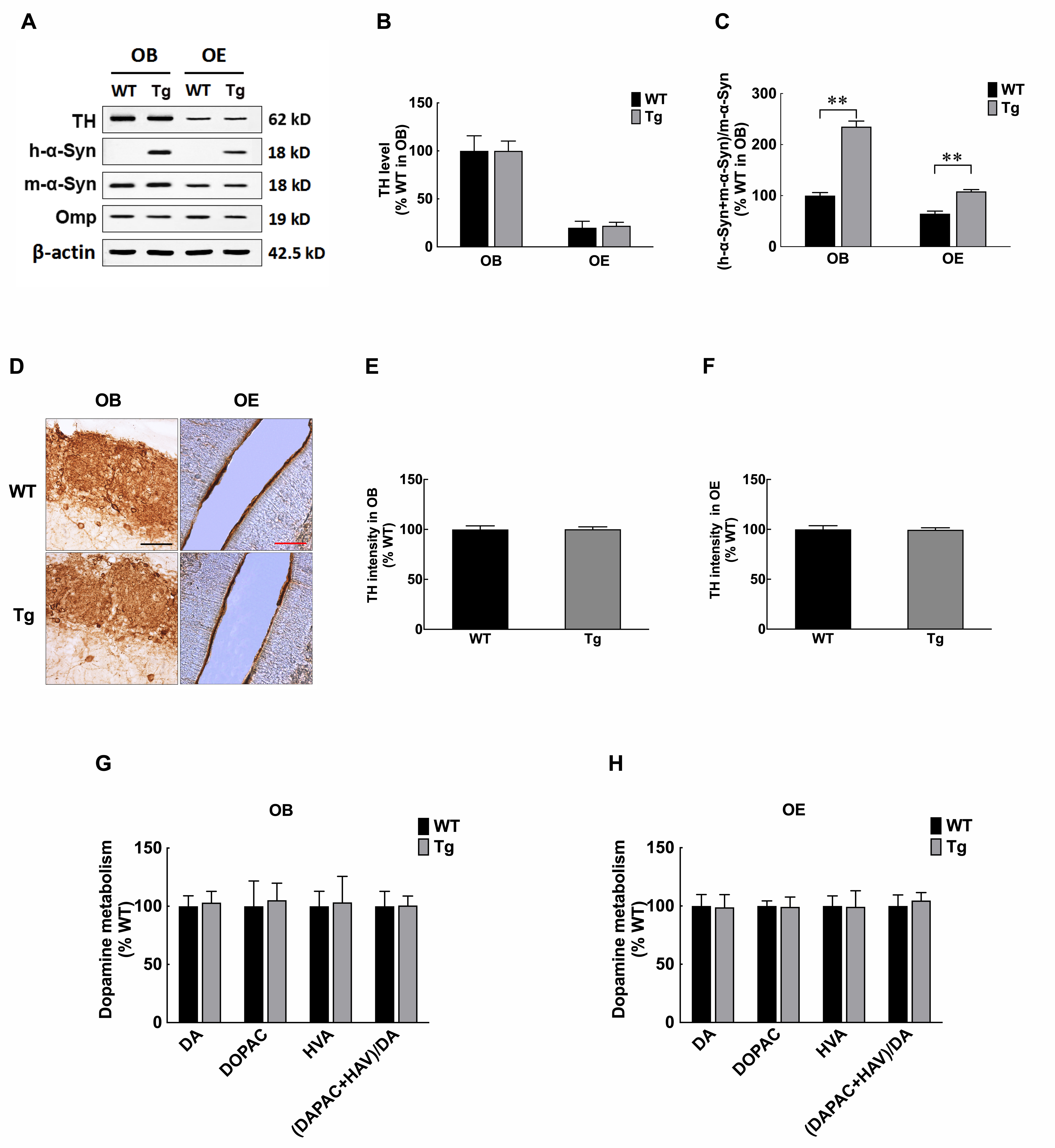

Supplement: Supplementary file 2 — Figure S2 [file JCMM-26-5008-s005.jpg]

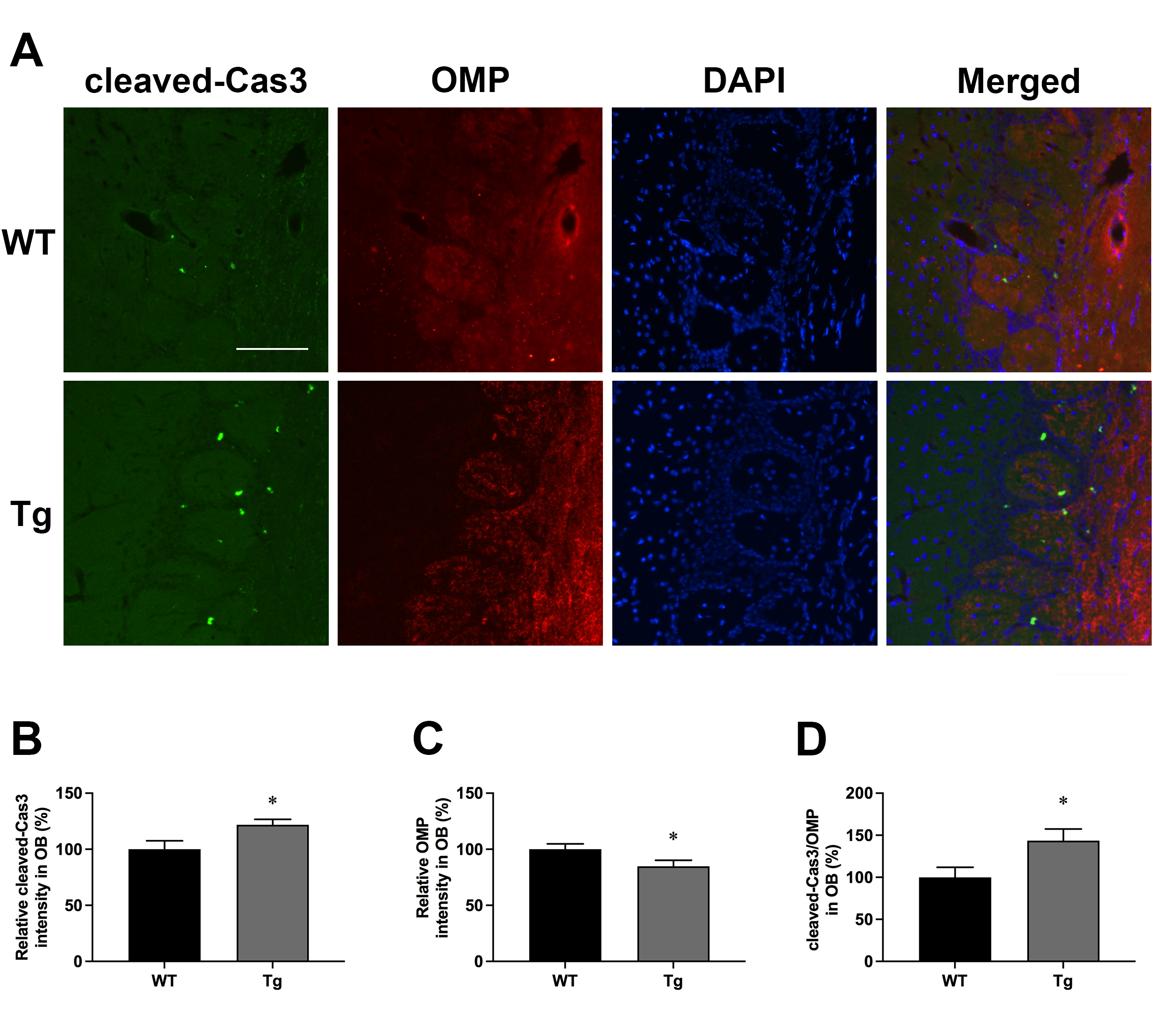

Supplement: Supplementary file 3 — Figure S3 [file JCMM-26-5008-s002.jpg]

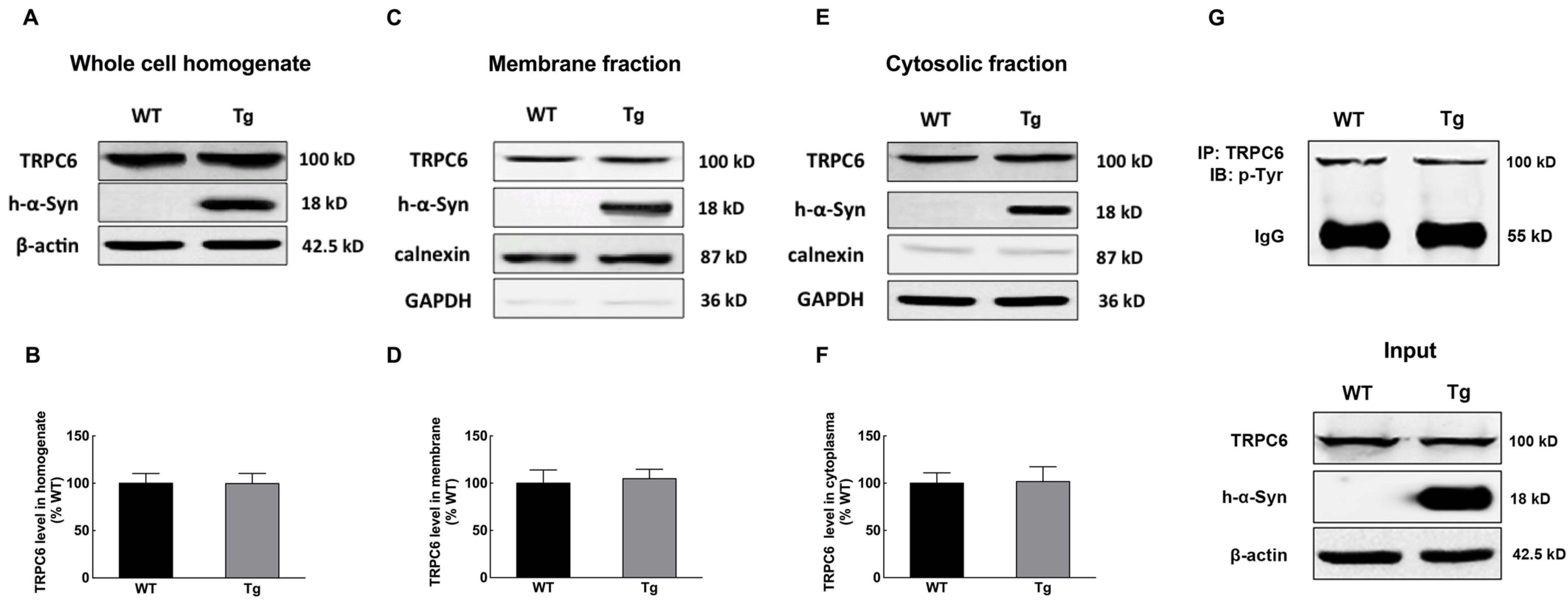

Supplement: Supplementary file 4 — Figure S4 [file JCMM-26-5008-s001.jpg]

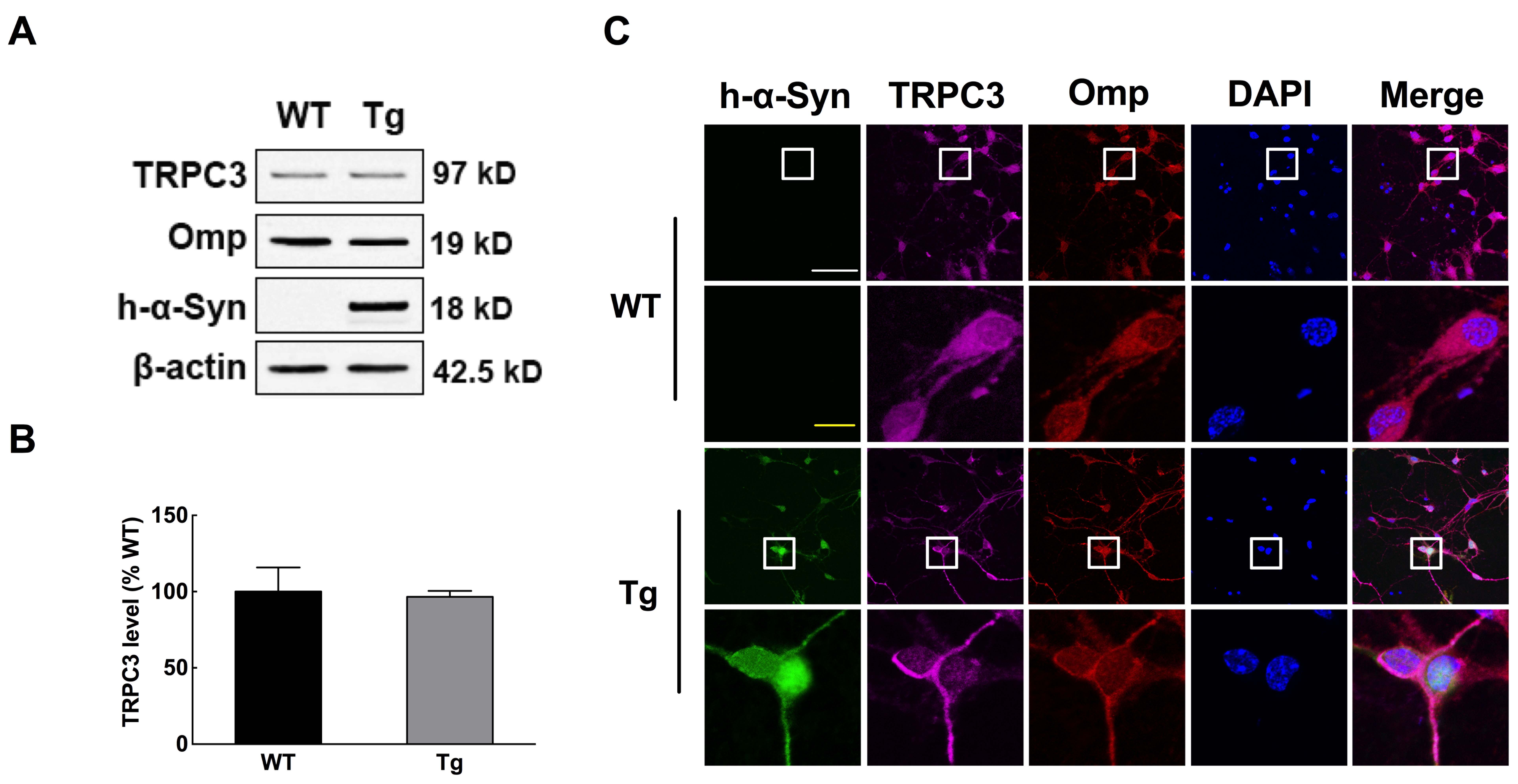

Supplement: Supplementary file 5 — Figure S5 [file JCMM-26-5008-s003.jpg]

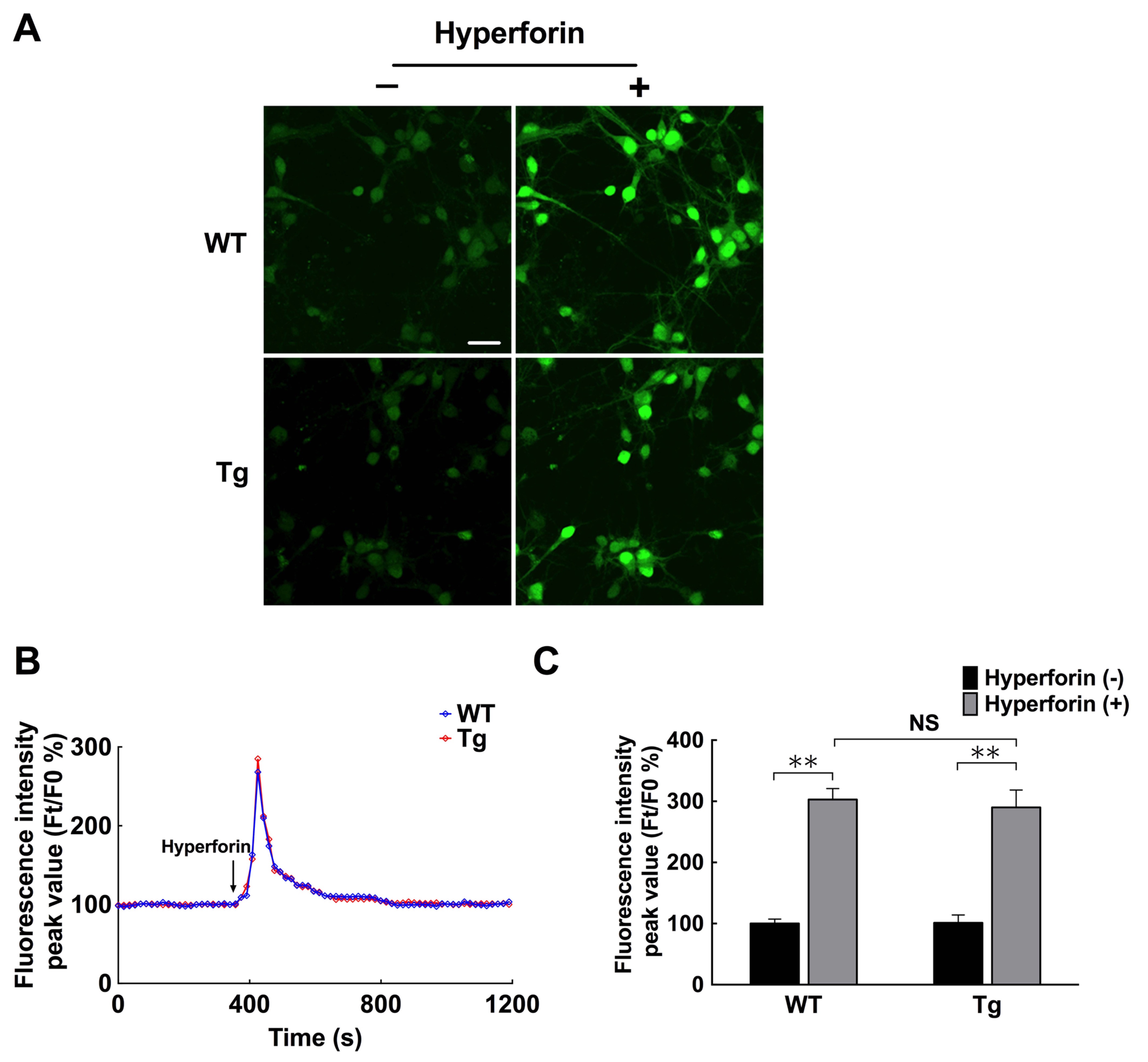

Supplement: Supplementary file 6 — Figure S6 [file JCMM-26-5008-s004.jpg]
